# In the Name of Family Medicine: A Nationwide Survey of Registered Names of Family Medicine Clinics in Taiwan

**DOI:** 10.3390/ijerph17114062

**Published:** 2020-06-07

**Authors:** Ya-An Liu, Sally Cheng, Ya-Chuan Hsu, Po-Chin Yang, Hsiao-Ting Chang, Ming-Hwai Lin, Tzeng-Ji Chen, Li-Fang Chou, Shinn-Jang Hwang

**Affiliations:** 1 Department of Family Medicine, Taipei Veterans General Hospital, No. 201, Sec. 2, Shi-Pai Road, Taipei 112, Taiwan; a3786923@gmail.com (Y.-A.L.); ych97160@gmail.com (Y.-C.H.); michael00557@gmail.com (P.-C.Y.); htchang2@vghtpe.gov.tw (H.-T.C.); minghwai@gmail.com (M.-H.L.); sjhwang@vghtpe.gov.tw (S.-J.H.); 2 Dr. Jiang’s Clinic, No.264, Wan-Da Road, Taipei 108, Taiwan; drpompon@gmail.com; 3 School of Medicine, National Yang-Ming University, No. 155, Sec. 2, Linong Street, Taipei 112, Taiwan; 4 Big Data Center, Department of Medical Research, Taipei Veterans General Hospital, No. 201, Sec. 2, Shi-Pai Road, Taipei 112, Taiwan; 5 Department of Public Finance, National Chengchi University, Taipei 116, Taiwan; lifang@nccu.edu.tw

**Keywords:** family medicine, clinics, names, professional identity, Taiwan

## Abstract

Family medicine is officially a specialty, but is often not regarded as a specialty by the general public. Past studies have usually investigated the opinions of medical students and resident physicians regarding family medicine, whereas few have focused on practicing family physicians themselves, especially in terms of analyzing how they represent themselves. This study aimed to investigate the patterns of clinic names to better apprehend whether general practitioners see themselves as being on an equal footing with other medical specialists. The registered names, medical specialties, and levels of urbanization of all clinics of Western medicine in Taiwan were collected. For clinics of each specialty, we examined whether their names contained the corresponding specialty designation. For example, a family medicine clinic was checked to determine whether its name contained the term “family medicine” or its abbreviation. The naming of family medicine clinics was then compared with that of clinics with other specialties. Of the 9867 Western medicine clinics included in this study, two-thirds (*n* = 6592) were single-specialty clinics. In contrast to the high percentages of single-specialty clinics of other specialties with specialty-containing names (97.5% for ophthalmology, 94.8% for dermatology, and 94.7% for otolaryngology), only 13.3% (132/989) of the family medicine clinics had such names. In addition, the urban family medicine clinics had a higher proportion (15.2%, 74/487) of specialty-containing names than the suburban (12.6%, 44/349) and rural family medicine clinics (9.2%, 14/153). Overall, a low percentage of family medicine clinics in Taiwan included “family medicine” in their names. This issue of professional identity deserves further qualitative investigation.

## 1. Introduction

Family medicine officially became a medical specialty around thirty years ago in many countries, such as the United States and Canada [1,2]. Family medicine specialists, who are generally known as “family physicians” or “family doctors,” provide comprehensive primary healthcare to patients and act as gatekeepers to the healthcare system. Therefore, family physicians are sometimes regarded as generalists as opposed to specialists in a narrow sense [3,4]. At the same time, in some countries where people are not required to register with family physicians, primary healthcare can also be provided by other specialists [5,6]. Because of their ambiguous recognition as specialists amongst society in general, the professional identity of family physicians remains a subject of research [7,8,9,10]. Past studies have usually investigated the opinions of medical students and resident physicians [11,12,13,14] regarding family medicine as their career choice, whereas few have focused on practicing family physicians themselves, especially in terms of analyzing how they represent themselves. The registered names of clinics, which are regarded as important means by which clinics can promote themselves, build their brands, and enhance their advertisements, can provide a clue about how physicians represent themselves by their branding.

In the current study, we aimed to investigate the patterns of registered names for family medicine clinics in comparison with those of clinics with other specialties in Taiwan. The results might provide evidence as to whether family physicians see themselves as being on an equal footing with other medical specialists. 

## 2. Materials and Methods 

### 2.1. Data Collection

We obtained the complete datasets regarding all healthcare facilities and healthcare personnel released by Taiwan’s Ministry of Health and Welfare from Taiwan’s Open Government Data platform (https://data.gov.tw/) in August 2016. These datasets provided information regarding healthcare facilities and their personnel, including the registered clinic name, address, type of ownership, specialties of the facility, the specialties of the physicians, and the headcounts of various types of medical personnel in each facility.

### 2.2. Study Design and Data Extraction

In this study, we constructed a Microsoft Excel worksheet and extracted the information of all 10,847 clinics of Western medicine, as of August 2016, from the aforementioned datasets regarding healthcare facilities and healthcare personnel. We excluded dental clinics and outpatient clinics of hospitals. Local health centers were also excluded because they were owned by the government. None of the words of any specialties appeared in the names of local health centers. Some specialties that are not conventionally suitable for clinics, e.g., nuclear medicine, pathology, radiology, radiation oncology, occupational medicine, anesthesiology, and emergency medicine, were also excluded in this study. Ultimately, 9867 clinics from the total of 10,847 clinics of Western medicine in Taiwan were included in this study.

We defined the clinics that employed physicians who all had the same specialty as single-specialty clinics; otherwise, they were defined as multi-specialty clinics. We stratified the single-specialty clinics and multi-specialty clinics by the main specialty that was registered by the clinic. We then reviewed the registered clinic name of all the clinics to find out whether or not each clinic’s own specialty was contained in its name. The specialty was regarded as being contained in the name if the full term or any type of abbreviation for the specialty was included in the clinic name. For example, clinic names that included the terms “family medicine”, “family”, and “FM” were all regarded as including the family medicine specialty.

In addition, we focused on the clinics with the single specialty of family medicine and classified them by urbanization level according to the urbanization stratification of Taiwan’s 368 townships determined by Taiwan’s National Health Research Institutes. All the townships in Taiwan were categorized into 7 levels by industrialization, demographic characteristics, and the distribution of medical resources [15]. We grouped Levels 1 and 2 as urban areas, Levels 3 and 4 as suburban areas, and Levels 5 to 7 as rural areas.

### 2.3. Statistical Analysis

Descriptive statistics were generated. The computations were performed by using Microsoft Excel 2019.

## 3. Results

### 3.1. Characteristics of Clinics of Western Medicine in Taiwan

**Figure 1:**
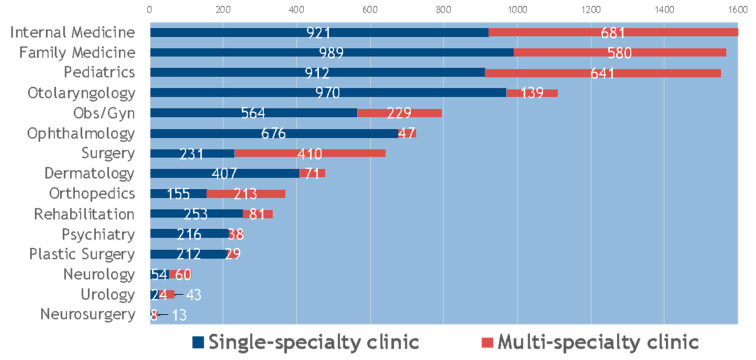
Numbers of single-specialty and multi-specialty clinics, stratified by specialty.

As shown in Figure 1, of the 9867 Western medicine clinics included in this study, the highest number of clinics specialized in internal medicine (16.2%, 1602/9867), followed by family medicine (15.9%, 1569/9867), pediatrics (15.7%, 1553/9867), and otolaryngology (11.2%, 1109/9867). The majority of these clinics (66.8%, 6592/9867) were single-specialty clinics.

### 3.2. Clinic Names that Included Their Own Specialty

**Figure 2:**
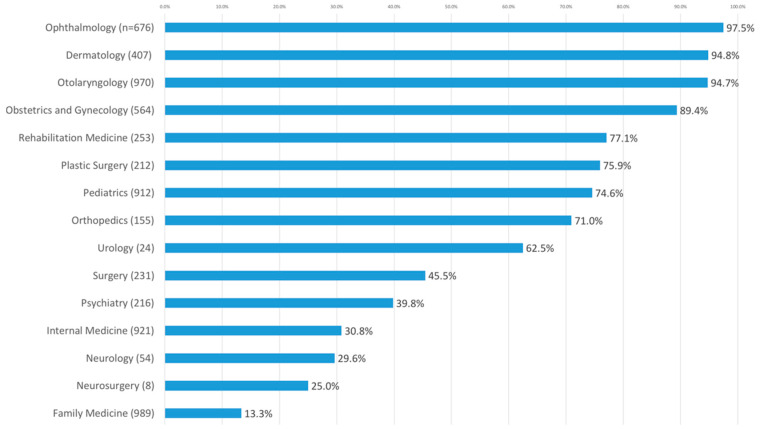
Percentages of single-specialty clinics containing their specialty in their registered clinic names.

Among the single-specialty clinics, those whose registered names included their own specialty were topped by those specializing in ophthalmology (97.5%, 659/676), followed by dermatology (94.8%, 386/407), otolaryngology (94.7%, 919/970), and obstetrics and gynecology (89.4%, 504/564), as shown in Figure 2. In contrast, the clinics specializing in family medicine (13.3%, 132/989) had the lowest rate of including their specialty in their names, followed by those specializing in neurosurgery (25.0%, 2/8), neurology (29.6%, 16/54), and internal medicine (30.8%, 284/921).

### 3.3. Single-Specialty Clinic Names Including a Term for “Family Medicine”

Among the 989 single-specialty clinics of family medicine included in this study, urban clinics had a higher rate (15.2%, 74/487) of including a term indicating “family medicine” in their clinic names, followed by suburban (12.6%, 44/349) and rural clinics (9.2%, 14/153), as shown in Table 1.

## 4. Discussion

### 4.1. Principle Findings

This study could provide a picture of the issue of professional identity faced by family physicians in Taiwan. In this study, 9867 clinics from the total of 10,847 clinics of Western medicine in Taiwan were included. According to the study results, most of the investigated Western medicine clinics in Taiwan (66.8%, 6592/9867) were single-specialty clinics. In contrast to the high percentages of single-specialty clinics with other specialties that had specialty-containing names (97.5% for ophthalmology, 94.8% for dermatology, and 94.7% for otolaryngology), the family medicine clinics (13.3%, 132/989) were the least likely to contain the specialty in their registered clinic names. In addition, urban family medicine clinics had a higher proportion of specialty-containing names (15.2%, 74/487) than did suburban (12.6%, 44/349) and rural family medicine clinics (9.2%, 14/153).

### 4.2. Features of Registered Clinic Names in Taiwan

The total population of Taiwan is about 23 million people, nearly all of whom are covered under the National Health Insurance system, which offers universal and comprehensive healthcare that is readily available at a very affordable cost [16]. Patients in Taiwan can visit clinics of any specialty to receive healthcare without referral and are not required to register with family physicians. There are more than 10,000 Western medicine clinics providing primary healthcare [6]. Under great pressures of competition, these clinics have made efforts to see as many patients as possible to increase their profits [16]. As such, clinics seek ways to stand out from other clinics in order to attract their target patients or clients. Relatedly, the registered names of clinics are regarded as important means by which clinics can promote themselves, build their brands, and enhance their advertisements [17].

According to our study results, some clinics with distinctive specialties, which have obvious targeted patients or clients, e.g., ophthalmology, dermatology, and otolaryngology, were more willing to indicate their specialties by including the specialties in their names. In contrast, more than sixty percent of psychiatrists did not use any words that were related to psychiatry in their clinic names. Previous studies have also reported that psychiatry clinics often refrain from including “psychiatry” or other relevant words in their names so as to avoid stigmatizing their patients [18]. A similar phenomenon has also been reported for palliative care units, which often avoid the terms “palliative” and “hospice” [19].

As opposed to other biomedical specialties, for example, eyes for ophthalmology and the skin for dermatology, family medicine does not have an organ that is strongly related to the specialty. Perhaps relatedly, while the number of family medicine clinics was the second highest among the clinics included in this study, words that indicated “family medicine” were least likely to be mentioned in their registered clinic names. Furthermore, family medicine practices in urban areas were more likely to include a term indicating “family medicine” in their clinic names than those in suburban and rural areas, although the differences were not great.

### 4.3. The Branding of Family Medicine 

Family medicine was officially recognized as a medical specialty in the United States in 1969 [20]. Meanwhile, more than thirty years have passed since family medicine was approved as a specialty by Taiwan’s Ministry of Health and Welfare and an association of family medicine was first founded in Taiwan [21]. However, there is still some controversy as to whether family medicine is truly a specialty or just a discipline for generalists [3,4,22]. Misconceptions about the practice of family medicine, which were created and reproduced in societal contexts amid the encouragement of the practice of medical specialties, might contribute to the current common view that family medicine does not enjoy the image, respect, and credibility it deserves [23,24]. Even in medical school education, the professional identity of family medicine might be less respected than those of other medical specialties. Less interesting, lower prestige, and intellectually less challenging were reported to be among the perceptions and attitudes of medical students toward family medicine [25,26]. We could speculate that these factors have all contributed to the fact that many family physicians have chosen to exclude the term “family medicine” in their clinic names.

In addition, poor general public recognition may also greatly contribute to this phenomenon, as the idea of having a family medicine doctor is still quite new to Taiwanese, and many people are not sure exactly what medical services family medicine can provide. Hence, in order to decrease confusion, many family medicine clinics might decide to exclude terms indicating “family medicine” from their names. However, the slight differences between urban and suburban and rural clinics in terms of including “family medicine” in their clinic names might be due to the fact that in rural areas where medical providers are scarce, many clinics offer primary healthcare regardless of their specialties, as they are often the only medical provider in the whole region. Hence, advertisements aimed at making one’s clinic stand out from other clinics may not have seemed necessary in many cases to rural clinics as compared to urban clinics.

Despite the aforementioned misconceptions, the important characteristics of family medicine, e.g., the continuity of healthcare, the comprehensive approach, the physician-patient relationship, and the spirit of cooperation, should be emphasized and promoted [27,28,29], especially by family physicians providing primary healthcare and improving public health. Moreover, in order to contribute to the professional identity of family medicine, good family medicine role models and training programs should be provided during the medical education of physicians [30,31].

### 4.4. Limitations

There were some limitations to our study. First, this was a cross-sectional study. Longitudinal changes to clinic names thus need to be further surveyed. Second, although some clinics had no specialties registered in the healthcare system, this did not necessarily mean that there were no specialists of any kind employed in these clinics. Third, we focused on clinics with the family medicine specialty in this study. Further surveys are needed, however, to clarify why many practitioners chose to exclude terms indicating “family medicine” from their clinic names or chose another specialty over family medicine. Furthermore, the real effects in terms of how the professional identities of different medical specialties influence registered clinic names also require further investigation.

## 5. Conclusions

According to this study, compared to clinics with other specialties, family medicine clinics in Taiwan have a low rate of including terms indicating their specialty in their names. This study could provide a picture of the issue of professional identity faced by family physicians in Taiwan, and this issue of professional identity deserves further qualitative investigation.

## Figures and Tables

**Table 1:** Single-specialty clinic names including a term for “family medicine” at different urbanization levels.

Urbanization Level	Number of Single-Specialty Clinics of Family Medicine	Number of Single-Specialty Clinics of Family Medicine Containing the Specialty in the Clinic Name
Urban	487 (100%)	74 (15.2%)
Suburban	349 (100%)	44 (12.6%)
Rural	153 (100%)	14 (9.2%)
Total	989 (100%)	132 (13.3%)

## References

[1] Future of Family Medicine Project Leadership Committee (2004). The future of family medicine: a collaborative project of the family medicine community. Ann. Fam. Med..

[2] Bailey T. (2007). Is family medicine a specialty? Yes. Can. Fam. Physician.

[3] Stein H.F. (2006). Family medicine’s identity: being generalists in a specialist culture?. Ann. Fam. Med..

[4] Elzinga H. (2005). A new identity for family medicine: physicians for the underserved. Fam. Pract. Manag..

[5] Song Y.J. (2009). The South Korean health care system. Jpn. Med. Assoc. J..

[6] Wu T.Y., Majeed A., Kuo K.N. (2010). An overview of the healthcare system in Taiwan. London J. Prim. Care (Abingdon).

[7] Rodríguez C., Pawlikowska T., Schweyer F.X., López-Roig S., Bélanger E., Burns J., Hugé S., Pastor-Mira M.Î., Tellier P.-P., Spencer S. (2014). Family physicians’ professional identity formation: a study protocol to explore impression management processes in institutional academic contexts. BMC Med. Educ..

[8] Carney P.A., Waller E., Eiff M.P., Saultz J.W., Jones S., Fogarty C.T., Corboy J.E., Green L. (2013). Measuring family physician identity: the development of a new instrument. Fam. Med..

[9] Beaulieu M.D., Rioux M., Rocher G., Samson L., Boucher L. (2008). Family practice: professional identity in transition. A case study of family medicine in Canada. Soc. Sci. Med..

[10] Rodríguez C., Tellier P.P., Bélanger E. (2012). Exploring professional identification and reputation of family medicine among medical students: a Canadian case study. Educ. Prim. Care.

[11] Scott I., Wright B., Brenneis F., Brett-Maclean P., McCaffrey L. (2007). Why would I choose a career in family medicine? Reflections of medical students at 3 universities. Can. Fam. Physician.

[12] Ozcakir A., Yaphe J., Ercan I. (2007). Perceptions of family medicine and career choice among first year medical students: a cross-sectional survey in a Turkish medical school. Coll. Antropol..

[13] Hansen S.E., Mathieu S.S., Biery N., Dostal J. (2019). The emergence of family medicine identity among first-year residents: a qualitative study. Fam. Med..

[14] Ie K., Tahara M., Murata A., Komiyama M., Onishi H. (2014). Factors associated to the career choice of family medicine among Japanese physicians: the dawn of a new era. Asia Pac. Fam. Med..

[15] Liu C.Y., Hung Y.T., Chuang Y.L., Chen Y.J., Weng W.S., Liu J.S., Liang K.Y. (2006). Incorporating development stratification of Taiwan townships into sampling design of large scale health interview survey. J. Health Manag..

[16] Taiwan Ministry of Health and Welfare National Health Insurance Annual Statistical Report 2018. https://www.mohw.gov.tw/cp-4574-49817-2.html.

[17] Chu F.Y., Dai Y.X., Liu J.Y., Chen T.J., Chou L.F., Hwang S.J. (2018). A doctor’s name as a brand: a nationwide survey on registered clinic names in Taiwan. Int. J. Environ. Res. Public Health.

[18] Dai Y.X., Chen M.H., Chen T.J. (2016). Low prevalence of the use of the Chinese term for ‘psychiatry’ in the names of community psychiatry clinics: a nationwide study in Taiwan. Int. J. Soc. Psychiatry.

[19] Dai Y.X., Chen T.J., Lin M.H. (2017). Branding palliative care units by avoiding the terms “palliative” and “hospice”: a nationwide study in Taiwan. Inquiry.

[20] Kahn N.B., Schmittling G., Ostergaard D., Graham R. (1996). Specialty practice of family practice residency graduates, 1969 through 1993: a national study. JAMA.

[21] Taiwan Association of Family Medicine Association Historiography. https://www.tafm.org.tw/ehc-tafm/s/w/Association/article/e437d4cf75184b89be4221205c4d040e.

[22] Brown C. (2018). Recognition of family physicians as experts rather than gatekeepers requires “cultural shift”. CMAJ.

[23] López-Roig S., Pastor M.Á., Rodríguez C. (2010). The reputation and professional identity of family medicine practice according to medical students: a Spanish case study. Aten. Primaria.

[24] Raghavendran S., Inbaraj L.R. (2018). Do family physicians suffer an identity crisis? A perspective of family physicians in Bangalore city. J. Family Med. Prim. Care.

[25] Selva Olid A., Zurro A.M., Villa J.J., Hijar A.M., Tuduri X.M., Puime Á.O., Dalmau G.M., Alonso-Coello P., for the Universidad y Medicina de Familia Research Group (UNIMEDFAM) (2012). Medical students’ perceptions and attitudes about family practice: a qualitative research synthesis. BMC Med. Educ..

[26] Wendling A.L., Wudyka A.E., Phillips J.P., Levine D.L., Mulhem E., Neale A.V., Morley C.P. (2016). RU4PC? Texting to quantify feedback about primary care and its relationship with student career interest. Fam. Med..

[27] Wright B., Scott I., Woloschuk W., Brenneis F., Bradley J. (2004). Career choice of new medical students at three Canadian universities: family medicine versus specialty medicine. CMAJ.

[28] Kim K.Y., Lim K., Park E.W., Choi E.Y., Cheong Y.S. (2016). Patients’ perceived quality of family physicians’ primary care with or without ‘Family Medicine’ in the clinic name. Korean J. Fam. Med..

[29] Premji K., Upshur R., Légaré F., Pottie K. (2014). Future of family medicine: role of patient-centred care and evidence-based medicine. Can. Fam. Physician.

[30] Vanasse A., Orzanco M.G., Courteau J., Scott S. (2011). Attractiveness of family medicine for medical students: influence of research and debt. Can. Fam. Physician.

[31] Gladu R., Nieman L.Z., Goodrich T.J., Groff J.Y., Velasquez M.M. (2002). Family medicine month in a university-based program: establishing a family medicine identity. Fam. Med..

